# Investigating the Role of P311 in the Hypertrophic Scar

**DOI:** 10.1371/journal.pone.0009995

**Published:** 2010-04-09

**Authors:** Jianglin Tan, Xu Peng, Gaoxing Luo, Bing Ma, Chuan Cao, Weifeng He, Shunzong Yuan, Shirong Li, John A. Wilkins, Jun Wu

**Affiliations:** 1 State Key Laboratory for Trauma, Burn and Combined Injury, Institute of Burn Research, Southwest Hospital, Third Military Medical University, Chongqing, China; 2 Chongqing Key Laboratory for Disease Proteomics, Chongqing, China; 3 Department of Plastic Surgery, First Affiliated Hospital of Chengdu Medical College, Chengdu, Sichuan Province, China; 4 Department of Plastic Surgery, Southwest Hospital, Third Military Medical University, Chongqing, China; 5 Manitoba Centre for Proteomics and Systems Biology, Department of Internal Medicine, University of Manitoba, Winnipeg, Canada; Tufts University, United States of America

## Abstract

The mechanisms of hypertrophic scar formation are not fully understood. We previously screened the differentially expressed genes of human hypertrophic scar tissue and identified P311 gene as upregulated. As the activities of P311 in human fibroblast function are unknown, we examined the distribution of it and the effects of forced expression or silencing of expression of P311. P311 expression was detected in fibroblast-like cells from the hypertrophic scar of burn injury patients but not in peripheral blood mononuclear cells, bone marrow mesenchymal stem cells, epidermal cells or normal skin dermal cells. Transfection of fibroblasts with P311 gene stimulated the expression of alpha-smooth muscle actin (α-SMA), TGF-β1 and α1(I) collagen (COL1A1), and enhanced the contraction of fibroblast populated collagen lattices (FPCL). In contrast, interference of fibroblast P311 gene expression decreased the TGF-β1 mRNA expression and reduced the contraction of fibroblasts in FPCL. These results suggest that P311 may be involved in the pathogenesis of hypertrophic scar via induction of a myofibroblastic phenotype and of functions such as TGF-β1 expression. P311 could be a novel target for the control of hypertrophic scar development.

## Introduction

Hypertrophic scars are a common problem after an injury. Clinically they present as erythematous or elevated firm plaques that remain confined to the damaged area. However if the formation of hypertrophic scar occurs in organs such as the lungs, liver, kidneys, joints, and skin, it can interfere with organ function, and ultimately result in organ failure. Even with the increasing knowledge of wound healing, hypertrophic scars are still difficult to prevent and to treat [1].

The formation of hypertrophic scar represents a dysregulated response to cutaneous wound healing and is characterized by inflammation, excessive proliferation of fibroblasts, and abnormal deposition of extracellular matrix (ECM) proteins [2]. Despite the fact that intrinsic differences between normal skin fibroblasts and hypertrophic scar myofibroblasts have been demonstrated by many studies, the underlying mechanisms promoting scar formation remain to be elucidated.

We previously screened for genes that were differentially expressed in hypertrophic scar and normal skin tissues of burn patients using gene microarrays and identified 97 differentially expressed genes. Among these P311 gene expression increased conspicuously in hypertrophic scar tissue [3].

The P311 gene is mapped to the long arm of chromosome 5 and the protein it encodes for does not belong to any known protein family [4]. The N-terminus of this 8 kDa protein contains a PEST domain that is highly conserved among human, murine and chicken proteins. This domain is a binding site of the ubiquitin/proteasome pathway which is involved in the regulation of expression of transcription factors, cytokines and signal factors [5], [6].

Pan et. al. reported that P311 protein and mRNA could be only found in myofibroblasts present in human wounds. Moreover, in their study the expression of P311 was associated with downregulation of TGF-β1 and collagen type I expression in the murine cell lines NIH 3T3 and C3H10T1/2 [7]. However, further study of the possible role(s) of P311 in human fibroblast function is required as hypertrophic scar is a unique feature of humans which is absent in other species including mice [8], [9], [10]. Therefore, in the present study, we examined the effects of forced and silenced P311 gene expression on human fibroblasts functions in vitro.

## Materials and Methods

### Patient Inclusion Criteria

Hypertrophic scar patients were selected according to the Vancouver Scar Scale (VSS) ranging from 10 score to 13 score [11]. 1.Tissues were collected within 1 year of the burn injury; 2. clinical manifestations: scar exhibited obvious hyperemia, red appearance, and obvious hypertrophy; patient experienced pruritus, pain, and dysesthesia of scar; local growth of the scars in the burned area; 3. there was no concomitant disease or history of use of immunosuppressants; 4. before surgery. All the patients were informed of the purpose and procedure of this study and agreed to offer their tissue specimens. The written consent was obtained from all participants involved in this study. All the protocols were approved by the Ethic Committee of Southwest Hospital, Chongqing, China.

### Preparation of Scar Specimens

All the hypertrophic scar specimens and the normal skin specimens were obtained from the same patients who underwent orthopedic surgery at the Institute of Burn Research,and the Department of Plastic and Reconstructive Surgery of Southwest Hospital. The specimens were either washed with PBS and subjected to immediate cell isolation or fixed with saturated trinitrophenol solution and storage in liquid nitrogen.

### Primary cell culture

As previously described [12], fibroblasts were from normal skins (Sk) and hypertrophic scars (Sc). Skin or hypertrophic scar tissue was cut into 0.5 cm^3^ pieces using a pair of scissors, and the epidermis and dermis were isolated by digestion with 0.25 g/l Dispase II (Sigma, USA). The dermal tissue was minced and digested thoroughly with 30 volumes of 200 U/ml collagenase I solution (Sigma, USA) (200 U collagenase I solution in 1 ml PBS) at 37°C for 2 hours, followed by cell collection by centrifugation. The cells were then cultured in DMEM medium containing 10% calf serum (Hyclone, USA) at 37°C in air containing 5% CO_2_. The cells were passaged 4 to 9 times and used in the following experiments. Epidermal cells (Ep) were from normal skins. Skin tissue was cut into 1 cm^3^ pieces using scissors and digested with 2.5 g/l Dispase II (Sigma, USA) at 4°C overnight. The epidermal tissue was collected to be digested by 2.5 g/l trypsin (Sigma, USA) containing 0.2 g/l EDTA·Na_2_ (Sigma, USA) for 5 minutes. And the epidermal cells were then cultured in K-SFM medium (Gibco, USA) at 37°C in air containing 5% CO_2_. Peripheral blood monocytes (PBM) were from the healthy peripheral blood. The 10 ml peripheral blood was diluted by 10 ml PBS, followed by adding into 20 ml Fercoll (1.073 g/ml) (TBD, China) gently. The cell suspension was then centrifuged at 900 g for 20 minutes at 20°C. The cells in up-interface were harvested to be diluted by 20 ml PBS. We got the peripheral blood monocytes precipitation after centrifuge the suspension at 700 g for 10 minutes at 20°C. Bone marrow mesenchymal stem cells (mMSC) were from healthy bone marrow and isolated with the same protocol as that for peripheral blood monocytes. The bone marrow mesenchymal stem cells were identified by FACS on the cell markers of CD105 (using FITC-labeled anti-CD105 antibody, 1∶50) and of CD34 (using PE-labeled anti-CD34 antibody, 1∶10) (Pierce, USA). The corresponding controls were either FITC-IgG_2b_ (1∶1000) or PE-IgG_1_ (1∶1000).

### Preparation of replication defective adenovirus expression vector containing human P311 gene (P311)

The P311 plasmid was a kind gift from Professor Matthieu Levi-Srauss (Inserm U114, Chaire de Neuropharmacologie, College de France, 11, Place Marcelin Berthelot, 75005 Paris, France). pAdTrack-CMV plasmid and pAdEasy™xL Adenoviral Vector System were purchased from Stratagene (USA). The P311 plasmid was digested and ligated with T vector. After the recombinant was identified by sequencing, pAdTrack-CMV/pGEM T-Easy-P311 shuttle plasmid was constructed and transfected into competent *E. coli* DH5α (ATCC, USA), followed by collection of rigid clones. Then, pAdTrack-CMV/pGEM T-Easy-P311 shuttle plasmid and adenovirus genome-containing pAdEasy-P311 plasmid underwent homologous recombination in *E. coli* BJ5183, and adenovirus plasmid expressing the target gene was screened. The virus was packaged and amplified in AD-293 cells, and the virus titer was determined by means of Plaque Forming Unit(PFU).

### Transduction of fibroblasts with replication defective adenovirus expression vector

Fibroblasts were digested with 0.25 g/l trypsin, and inoculated into 75 cm^2^ culture flasks at a density of 1×10^6^ cells in 10 ml DMEM medium containing 10% calf serum. Upon 80% cell confluence, the medium was discarded, and the cells were washed with 0.1 M PBS and antibiotic-free, serum-free DMEM medium. Then, 1×10^9^ PFU/ml virus stock suspension was added to cover the cells on the culture flask bottom. The cells were cultured for 1 hour at 37°C in air containing 5% CO_2_, and then the virus suspension was removed, followed by the addition of DMEM medium containing 10% calf serum, and cultured in an incubator. At the indicated time points, the cultures were microscopically examined for the expression of green fluorescent protein using an inverted microscope (Olympus, Japan).

### Preparation of the model of fibroblast populated collagen lattice (FPCL) and measurement of fibroblast contraction in FPCL Model

The FPCL model was used to observe the contractile ability of fibroblasts. As described previously [13], soluble collagen was extracted from the tails of SD rats. The cell suspension (the final concentration: 5×10^5^ cells/ml), calf serum (the final concentration: 10%), 5×DMEM, and collagen protein solution were mixed at a volume ratio of 1∶1∶2∶6 at 4°C and the pH of the mixture was adjusted to 7.2. The fibroblast/collagen protein suspension was added into culture dishes at a size of 35 mm in diameter, and maintained for 10 minutes at 37°C in air containing 5% CO_2_ until gel formation, followed by addition of 1 ml DMEM medium containing 10% calf serum and further cultured at 37°C in air containing 5% CO_2_. The diameter of FPCL was recorded every other day. The FPCL contraction index is calculated as follows [14]: CI = [1−(D/D_0_)^2^]×100% (CI: contraction index; D: diameter of collagen protein gel block; D_0_: initial diameter of gel block (35 mm)).

### Silencing of P311 in fibroblast by shRNA

This study used three P311 shRNAs, targeting different regions of the P311 transcript and one control shRNA contracted shRNA1, shRNA 2, shRNA 3 and control. The sequences of shRNA1 were 5′-GATCCGCCGCAAGAAGAACGATGAGTTCAAGACGCTCATCGTTCTTCTTGCGGTTTTTTGTCGACA-3′ and 3′-GCGGCGTTCTTCTTGCTACTCAAGTTCTGCGAGTAGCAAGAAGAACGCCAAAAAACAGCTGTTCGA-5′. shRNA2 sequences were 5′-GATCCGAACGATGAGACAAACGCTTTCAAGACGAGCGTTTGTCTCATCGTTCTTTTTTGTCGACA-3′ and 3′-GCTTGCTACTCTGTTTGCGAAAGTTCTGCTCGCAAACAGAGTAGCAAGAAAAAACAGCTGTTCGA-5′. shRNA3 sequences were 5′-GATCCGAAGAACGATGAGACAAACTTCAAGACGGTTTGTCTCATCGTTCTTCTTTTTTGTCGACA-3′ and 3′-GCTTCTTGCTACTCTGTTTGAAGTTCTGCCAAACAGAGTAGCAAGAAGAAAAAACAGCTGTTCGA-5′. And the sequences of the control shRNA were 5′-GATCCGACTTCATAAGGCGCATGCTTCAAGACGGCATGCGCCTTATGAAGTCTTTTTTGTCGACA-3′ and 3′-GCTGAAGTATTCCGCGTACGAAGTTCTGCCGTACGCGGAATACTTCAGAAAAAACAGCTGTTCGA-5′. These shRNA fragments were ligated with pGenesil-1 plasmid (Genesil, China). Primary human fibroblasts containing about 2×10^4^ cells were transfected with the 1 µg P311 shRNA plasmids using 3 µl FuGene 6 non-liposome transfection reagent (Roche, USA). Stable fibroblasts clones were selected with medium containing G418 (400 µg/ml) for 3 weeks and the expression levels of P311 protein were determined by Western blotting. Clones displaying satisfactory P311 gene suppression were selected for subsequent analysis. Scar fibroblasts expressing shRNA gene were divided into the following groups: 1. Sc (hypertrophic scar-derived fibroblast-like cells); 2. control (hypertrophic scar-derived fibroblast-like cells transfected with negative plasmid, served as control group); 3. shRNA1 (hypertrophic scar-derived fibroblast-like cells transfected with shRNA1 plasmid); 4. shRNA2 (hypertrophic scar-derived fibroblast-like cells transfected with shRNA2 plasmid); 5. shRNA3 (hypertrophic scar-derived fibroblast-like cells transfected with shRNA3 plasmid).

### Western Blotting

Cells were lysed using 1% protease inhibitor cocktail, 1% phenymethylsulphonyl fluoride and 1% sodium orthovanadate in RIPA lysis buffer (Santa, USA). The samples were scraped, collected and centrifuged at 10,000 g for 30 minutes. The supernatant was collected, and protein concentrations were determined by Bradford assay. Equal amounts of protein were mixed with reducing SDS sample buffer and boiled for 5 minutes before loading onto 10% SDS-PAGE gels. Electrophoresis was carried out under reducing conditions at 100 volts for 1.5 hours and the separated proteins were then transferred at 100 volts over 1.5 hours to a nitrocellulose (NC) membrane (GE, USA). The membrane was blocked with Tris-buffered saline (TBS) containing 5% non-fat powdered milk for 2 hours at room temperature and then incubated with rabbit anti-human P311 antibody (1∶1000) (Boster, China) at 4°C overnight. The blots were subsequently washed with TBS containing 1% Tween 20 for 4 times and then incubated with HRP-labeled goat anti-rabbit secondary antibody (1∶2000) (Boster, China) for 2 hours at room temperature. Proteins were washed with TBS containing 1% Tween 20 for 4 times again, and visualized using enhanced chemiluminescence (Pierce, USA) according to the manufacturer's instructions.

### RT-PCR

P311 mRNA expression was detected in skin- and hypertrophic scar-derived fibroblasts by RT-PCR, and the expression of α-SMA, TGF-β1, α1(I) collagen gene (COL1A1) mRNAs were detected in fibroblasts before and after P311 gene transduction. The primers for the genes were designed according to the sequence registered in Genbank, and synthesized by Shanghai Bioaisa Biotech Co. Ltd. Total RNA was extracted from cultured cells with TRIzol reagent (Gibco, USA). RT-PCR was performed as described previously. The RNA was made at 42°C for 15 minutes using AMV reverse transcriptase (Promega, USA) and oligo(dT) as a primer. The optimal reaction conditions were determined for each experimental group and primers. The primers for P311 were 5′-GCACATATGGTTTATTACCCAG-3′ and 5′-CAGGATCCTTAAAAAAAGTGGAGG-3′. The primers for α-SMA were 5′-GGTGATGGTGGGAATGG-3′ and 5′-TGGCTGGAACAGGGTCT-3′. The primers for TGF-β1 were 5′-CTGTGGCTACTGGTGCTGAC-3′ and 5′-CATAGATTTCGTTGTGGGTTTC-3′. The primers for α1(I) collagen gene(COL1A1) were 5′-TCTTTGAATCCTAGCCCATC-3′ and 5′-GGAGCACCTTTACAAGCAGT-3′. The primers for glyceraldehyde phosphodehydrogenase(GAPDH) were. 5′-CGTCTTCACCACCATGGAGA-3′ and 5′-CGGCCATCGCCACAGTTT-3′. Reaction conditions were 5minutes at 94°C and 35 cycles of 94°C for 30 seconds, 55°C for 30 seconds, and 72°C for 40 seconds. GAPDH was used as an internal control. PCR products were separated by electrophoresis on 1% agarose gels, stained with ethidium bromide, and photographed against ultraviolet light. Expression levels of target genes were normalized by concurrently measured GAPDH mRNA levels. The mRNA relative content can be showed by the ratio of its PCR and GADPH absorbency.

### Analysis of cell cycle by FACS

Cells were detached in 0.25 g/l trypsin and washed twice in ice-cold PBS. Followed by fixation in 70% ethanol in 4°C overnight, cells were washed 2 additional times in PBS, and then stained for 30 minutes at 37°C in 50 µg/ml propidium iodide (Sigma, USA) solution containing 200 µg/ml RNase A and 0.1% Triton-X-100. Samples were stored at 4°C until analysis by FACS (BD, USA).

### Statistical analysis

Measurement data were presented as means ± standard deviation (SD) and analyzed by one-way ANOVA test in conjunction with LSD test. The differences between multiple specimens were analyzed when necessary. P value less than 0.05 was considered as the statistical significance.

## Results

### The distribution of P311

**Figure 1:**
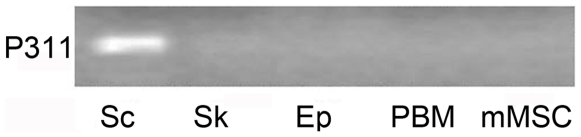
The expression of P311 in different cell types. Total RNA was isolated from cells and RT-PCR was performed to detect P311. The groups were designed as Sc (hypertrophic scar-derived fibroblast-like cells), Sk (skin-derived fibroblasts), Ep (epidermal cells), PBM (peripheral blood monocytes) and mMSC (bone marrow mesenchymal stem cells). Representative result is from 1 of 10 experiments.

The expression pattern of P311 mRNA was examined in several human cell types by means of RT-PCR. P311 was readily detectable in dermal fibroblast-like cells derived from hypertrophic scar tissue. However, it was not detected in the normal skin-derived dermal fibroblasts, epidermal cells, peripheral blood mononuclear cells or bone marrow mesenchymal stem cells (Fig. 1).

### The effect of P311 over expression on fibroblast functions

**Figure 2:**
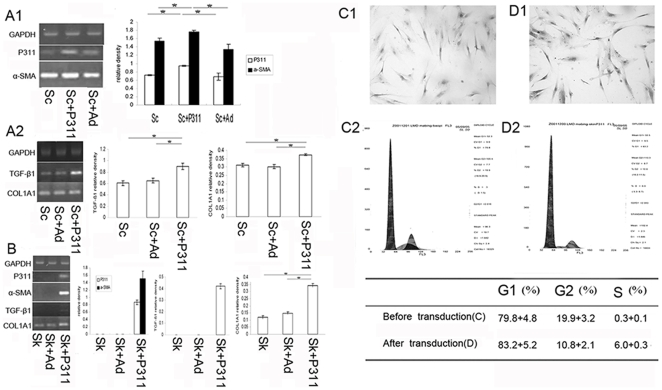
The effect of P311 on fibroblast functions after P311 gene upregulation. Total RNA was isolated from fibroblasts and RT-PCR was performed to compare α-SMA, TGF-β1 and α1(I) collagen gene(COL1A1) mRNA levels in different treated groups. (A) Hypertrophic scar-derived fibroblast-like cells were transduced with adenovirus P311 and cultured as described under materials and methods. The groups were designed as Sc (hypertrophic scar-derived fibroblast-like cells), Sc+Ad (hypertrophic scar-derived fibroblast-like cells +adenovirus void vector) and Sc+P311 (hypertrophic scar-derived fibroblast-like cells +P311). (B) Normal skin-derived fibroblasts were transduced with adenovirus P311. The groups were Sk (normal skin-derived fibroblasts), Sk+Ad (normal skin-derived fibroblasts+adenovirus void vector) and Sk+P311 (normal skin-derived fibroblasts+P311), * means P<0.01 between the two groups. (C, D) Transduction of P311 into normal skin-derived fibroblasts increased the S-Phase cell fraction as determined by FACS. (C) Control group (×200). (D) P311 transduced group (×200). A representative analysis of 3 experiments is shown.

The effects of transduction of the human P311 gene or its empty control virus into hypertrophic scar-derived or normal dermal fibroblasts were examined. We observed that normal skin-derived fibroblasts do not express α-SMA or TGF-β1 (Fig. 2B). Following transduction with a P311 expression vector, α-SMA, TGF-β1, and COL1A1 mRNAs were all increased (Fig. 2B). Moreover, the proportion of cells in S phase was increased from 0.3% to 6.0% following P311 expression (Fig. 2C and 2D).

In contrast hypertrophic scar-derived dermal fibroblast-like cells constitutively express α-SMA, TGF-β1, and COL1A1 (Fig. 2A1 and 2A2). However, the upregulation of P311 did increased the expression of α-SMA, TGF-β1, and COL1A1 mRNA.

**Figure 3:**
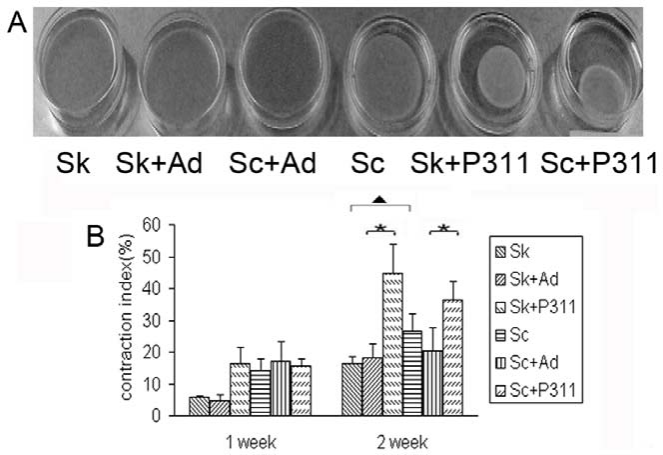
The contraction of collagen by fibroblasts. Normal skin- and hypertrophic scar-derived fibroblasts were transduced with recombinant adenovirus P311 as described in Materials and Methods Section. The groups were designed as Sk (normal skin-derived fibroblasts), Sk+Ad (normal skin-derived fibroblasts+adenovirus void vector), Sk+P311 (normal skin-derived fibroblasts+P311), Sc (hypertrophic scar-derived fibroblast-like cells), Sc+Ad (hypertrophic scar-derived fibroblast-like cells +adenovirus void vector and Sc+P311 (hypertrophic scar-derived fibroblast-like cells +P31). (A) Normal skin- and hypertrophic scar-derived fibroblasts were transduced with adenovirus P311, and cultured in a collagen lattice for 13 days. (B) P311 enhanced the contractile ability of fibroblasts obviously. The FPCL model was cultured for 1, 2, 3 and 4 weeks. The contraction index was calculated as previous described. * means p<0.01 as compared with the control group without P311. ▴ means p<0.01 between hypertrophic scar-derived fibroblast-like cells and normal skin-derived fibroblasts. A representative analysis of 3 experiments is shown.

The ability to induce collagen contraction is a characteristic feature of myofibroblasts [15]. A comparison of the contractile activities of normal skin-derived fibroblasts with those derived from hypertrophic scar indicated that the normal cells expressed lower activity (Fig. 3). Transduction of P311 into normal skin-derived fibroblasts markedly enhanced their ability to contract collagen gels (Fig. 3). Similarly transduction of P311 into hypertrophic scar derived fibroblast-like cells significantly enhanced their ability to contract the gels (Fig. 3).

### The suppression of P311 expression inhibited scar-derived fibroblast-like cells functions

The above observations suggested that P311 expression induces a number of the phenotypic properties observed in hypertrophic scar derived fibroblast-like cells. Furthermore as P311 expression was elevated in scar tissues, we tested the effects of inhibition of P311 expression on the properties of scar-derived fibroblast-like cells.

**Figure 4:**
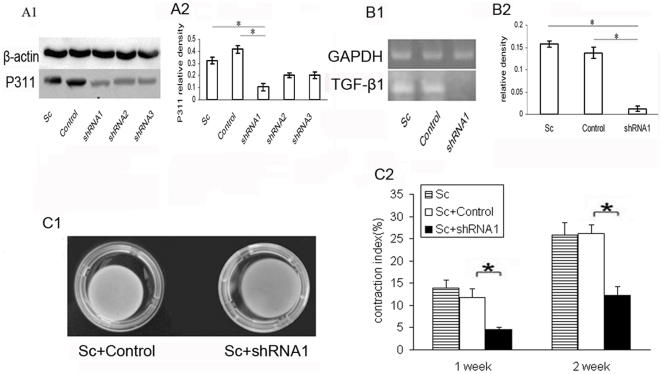
The expression of TGF-β1 by fibroblasts after P311 shRNA plasmid transfection. (A) Total protein was extracted from hypertrophic scar-derived fibroblast-like cells transfected with P311 shRNA plasmids. Western blotting demonstrated that shRNA1 plasmid was the most effective plasmid for silencing P311 protein expression in fibroblasts. * means P<0.01 between the two groups. (A1) The expression of P311 protein is demonstrated by Western blotting. (A2) The lowest expression of P311 protein was found in shRNA1 group. (B) Hypertrophic scar-derived fibroblast-like cells transfected with P311 shRNA plasmids showed lower expression of TGF-β1 than control. * means P<0.01 between the two groups. (B1) Total RNA was isolated from hypertrophic scar-derived fibroblast-like cells and RT-PCR was performed to determine TGF-β1 mRNA level. (B2) Histogram showed the downregulation of TGF-β1 mRNA level in P311 silenced hypertrophic scar-derived fibroblast-like cells. (C) Hypertrophic scar-derived fibroblast-like cells were transduced with shRNA of P311. The groups were Sc+control (hypertrophic scar-derived fibroblast-like cells + negtive vector (control)) and Sc+ shRNA1 (hypertrophic scar-derived fibroblast-like cells + P311 shRNA1). (C1) Hypertrophic scar-derived fibroblast-like cells were transfected with P311 shRNA plasmids and cultured in a collagen lattice for 2 weeks. (C2) Absence of P311 inhibited the contractile ability of hypertrophic scar-derived fibroblasts. * means p<0.01 considered as statistical significance as compared with the control group with negative shRNA plasmids (control). A representative analysis of 3 experiments is shown.

Transfection of scar-derived fibroblast-like cells with a shRNA targeting P311 (shRNA1) significantly lowered the expression levels of P311 protein as detected by western blot (Fig. 4A). These cells displayed reduced levels of TGF-β1 expression (Fig. 4B). Significantly, there was a partial loss of the contractile ability of fibroblast-like cells in the FPCL model following the suppression of P311 (Fig. 4C).

## Discussion

The formation of hypertrophic scar is an abnormal wound healing response after trauma. Histologically and clinically, hypertrophic scar is characterized by excessive fibroblast hypertrophy, accumulation of extracellular matrix and contracture [16], [17], [18], [19]. At the molecular level, TGF-β is considered to be the critical regulator involved in the formation of hypertrophic scar [20], [21]. However, there are currently no approaches based on this paradigm which completely prevent scar formation or treat existing scar [1], [20], [22]. This leads us to search for additional potentially unknown factors involved in hypertrophic scar formation, which might aid our understanding of hypertrophic scar formation. Such information might also suggest novel strategies for controlling scar formation.

Our previous microarray analysis revealed that P311 was highly upregulated in hypertrophic scar tissue [3]. Meanwhile, Pan et. al. showed that P311 was found in human wounds during healing and disappeared after wound healed [7]. Importantly, Pan also demonstrated that transfection of P311 into murine cell line NIH 3T3 or C3H10 T1/2 could significantly stimulate the differentiation of fibroblasts to myofibroblasts via a TGF-β1 independent signaling pathway. Significantly, these results were not obtained with human fibroblasts. The combination of our previous work [3] and that of Pan et. al. prompted us to explore the possible roles of P311 in human fibroblast function.

An examination of P311 mRNA expression in a number of human cell types revealed that P311 mRNA expression was only detected in hypertrophic scar-derived fibroblast-like cells. These results are consistent with the finding of immunohistochemical studies of Pan et. al. [7]. These observations suggest that P311 expression may be involved in normal and hypertrophic scar formation.

Our results show that the expression of P311 can induce the expression of α–SMA (Fig. 2B) in normal skin-derived fibroblasts. As α-SMA is a putative cell marker of myofibroblasts [23], this result suggests that P311 may induce the myofibroblast phenotype. These observations are consistent with those seen in murine fibroblasts [7]. However our current work also indicated that the forced expression of P311 was associated with increased levels of TGF-β1 and COL1A1 mRNA in both normal skin-derived and hypertrophic scar-derived fibroblast-like cells (Fig. 2A2 and 2B) relative to the corresponding control transductants. Conversely inhibition of P311 gene expression resulted in a reduction in the expression of TGF-β1. In addition, after P311 gene transduction, more fibroblasts entered into the S phase of cell cycle (Fig. 2D), which implies that P311 can stimulate the proliferation of fibroblasts. Our results indicate that P311 promotes the production of TGF-β and COL1A1 in human fibroblasts, and stimulates their proliferation and differentiation to myofibroblasts.

We tested the role of P311 in the contraction of fibroblasts in FPCL model in vitro, as myofibroblasts are believed to be responsible for the contraction of hypertrophic scar [24] and α–SMA positive myofibroblasts can cause the contraction of FPCL in vitro [13], [15]. Expression of P311 gene was associated with an enhanced level of contraction in normal and hypertrophic scar-derived fibroblasts (Fig. 3). On the other hand, suppression of P311 expression in hypertrophic scar-derived fibroblasts-like cells inhibited the capacity of these cells to mediated collagen contraction (Fig. 4C). These data indicate that P311 gene might be also involved in the contraction of hypertrophic scar.

TGF-β1 is considered as the key paracrine/autocrine growth factor in induction and maintenance of the hypertrophic scar. In addition the signaling pathway of TGF-β1 initiates a mechanistic cascade involving activation of fibroblast differentiation which promotes the synthesis of α–SMA and the extracellular matrix, mainly collagen I, with enhanced the contractile ability [1]. Therefore, we hypothesize that P311 induces fibroblast differentiation via enhancing the TGF-β1 signaling pathway in human hypertrophic scar.

Our results do not seem to be consistent with those of Pan et al which suggested that P311 induced TGF-β1-independent myofibroblast transformation. We do observe myofibroblast transformation but this occurs in the presence of up regulation of TGF-β1 and collagen. The former results were based on data obtained with murine fibroblasts. Hypertrophic scars appear to be unique to humans as no animals are known to form these lesions [8], [9], [10]. Furthermore, animal models including athymic mice, rats, hamster cheek pouch, guinea pigs and rabbit ear models present significant differences when compared with human hypertrophic scars suggesting that these may have different pathogenic mechanisms than human hypertrophic scar [25]. In our study, we employed human fibroblasts from normal skin and hypertrophic scar as the target cells which is consistent with the pathogenesis of human hypertrophic scar. The results of our study suggest that P311 may have different regulatory functions in fibroblasts of human origin compared with in murine fibroblasts.

In summary, our work for the first time shows the function of P311 gene in human fibroblasts, i.e., P311 gene can stimulate human fibroblast proliferation, induce myofibroblast phenotype, promote the expression of TGF-β1 and COL1A1, and enhance the contraction of human fibroblasts in FCPL. These data strongly indicate that P311 may be involved in the hypertrophic scar pathogenesis and contraction via enhancing the expression of TGF-β1. Further studies of P311 function may provide new insights into the molecular events of hypertrophic scar formation and possibly provide new approaches for the control of hypertrophic scar formation.
